# Understanding the context of healthcare utilisation for children under-five with diarrhoea in the DRC: based on Andersen behavioural model

**DOI:** 10.1186/s12913-022-07530-4

**Published:** 2022-02-04

**Authors:** Siyu Zou, Xinran Qi, Keiko Marshall, Maria Bhura, Rie Takesue, Kun Tang

**Affiliations:** 1 Vanke School of Public Health, Tsinghua University, Beijing, 100191 China; 2 School of Public Health, Peking University Health Science Center, Beijing, China; 3 School of Nursing, Capital Medical University, Beijing, China; 4 Division of Epidemiology, Dalla Lana School of Public Health, University of Toronto, Toronto, Canada; 5 Department of Paediatrics and Child Health, Aga Khan University, Karachi, Pakistan; 6 Health Section Programme Division, UNICEF Headquarters, New York, USA

**Keywords:** Andersen Behavioural model, Healthcare utilisation, Diarrhoea, DRC, Under five-year-old children

## Abstract

**Background:**

Diarrhoea is one of the leading causes of death among children under 5 years old in the Democratic Republic of the Congo (DRC). Despite positive effects on prognosis, there is limited literature about the healthcare-seeking behaviours of children with diarrhoea, especially in the DRC. This study used the Andersen Behavioural Model, a theoretical framework, which was commonly adopted to study healthcare utilisation, to investigate and predict factors associated with the use of healthcare to treat diarrhoea in the DRC.

**Methods:**

Data collected from 2626 under-five children with diarrhoea in the last 2 weeks from the Multiple Indicators Cluster Survey conducted by the National Institute of Statistics in 2017–2018, in collaboration with the United Nations Children’s Fund were used in this study. Both direct and indirect relationships among four latent variables: predisposing traits, enabling resources access, health needs, and health services use were measured using the structural equation modelling to test the Andersen behavioural model. The confirmatory Factor Analysis model was also modified based on the DRC context to explore this further.

**Results:**

The modified model had the goodness of fit index (GFI) of 0.972, comparative fit index (CFI) of 0.953 and RMSEA of 0.043 (95% CI: 0. 040, 0.047). Health needs (especially diarrhoea) had the largest positive direct effect on healthcare utilisation (standardized regression coefficient [β] = 0.135, *P* < 0.001), followed by “enabling resources” (β = 0.051, *P* = 0.015). Health needs also emerged as a mediator for the positive effect of predisposing on utilisation (indirect effect, β = 0.014; *P* = 0.009).

**Conclusion:**

Access to improved water and improved sanitation, as well as socioeconomic factors like household wealth, were significantly associated with health-seeking behaviours for diarrhoea treatment in the DRC. Besides, caregivers who own higher levels of educational attainments were more inclined to have positive health services uses during the treatments. Efforts are needed to enhance the oral rehydration therapy coupled with educating caregivers on its appropriate use.

**Supplementary Information:**

The online version contains supplementary material available at 10.1186/s12913-022-07530-4.

## Background

Sub-Saharan Africa has made essential progress in reducing child mortality [1], whereas the child mortality rate in the Democratic Republic of the Congo (DRC) remains high at 84.8 deaths per 1000 live births, far from achieving the Sustainable Development Goal (SDG) 3.2 which aims to reduce under-five mortality to 25 deaths per 1000 live births [2, 3]. The DRC is one of the largest, but most vulnerable [4] and poorest countries [5] in Sub-Saharan Africa (SSA). It has over 50% of the African continent’s water reserves while only 52% of the population has access to an improved water source and 29% has improved sanitation facilities with sustained efforts in rural areas [6]. Through 2018, it had the fifth-highest under-five mortality rate globally [7, 8] and diarrhoea was found to be one of the main leading causes of mortality amongst children under five years of age, accounting for 15% of deaths [9]. However, appropriate healthcare-seeking behaviours could efficiently decrease child morbidity and mortality [10] as studies from various countries suggest that healthcare-seeking behaviour for childhood illnesses is often uncommon, particularly in the low- and middle-income countries (LMICs) [11], especially for such a context where an estimated 465,000 children in the DRC die per year from preventable diseases like diarrhoea [8]. In other words, the poor healthcare utilisation status there could be tackled strategically.

Social scientists have emphasized that studies focusing on healthcare-seeking behaviours and utilisation of health services may provide a better understanding of factors with potential programmatic and political implications in improving the health status of individuals [12]. Many studies have shown differences in healthcare utilisation based on patients’ social characteristics [13]. Furthermore, diverse conceptual models have been developed and applied in explaining and specifying the interrelationships of the array of possible predictors of healthcare utilisation behaviour, and in guiding the conduct of analytic and evaluative research [14–17], including accessibility, availability, acceptability, affordability, adequacy, and appropriateness in final decision-making for healthcare seeking. Among them, the Andersen Behavioural Model (BM) is one of the most classic and well-acknowledged models as it is a multilevel model incorporating both individual and contextual determinants of health services use that has been the most widely accepted and adopted in developed countries [18–22] with convincing conclusions, whose variants have proven universal, as they have been successfully used when studying healthcare-seeking behaviour for periodontal health [23], stomatology [24–26], and mental illness [27] over time with many updated versions. Revisions of BM have been presented, and all suggest that health services use is a function of predisposing characteristics (P), enabling resources that facilitate access to health services (E), and health needs (N) [18]. In our study, P, E, and N at the individual level are instrumental for increasing healthcare-seeking behaviour and healthcare utilisation [22, 28]. Individual and community-level factors also determine the occurrence and outcome of diarrhoea [22, 29, 30].

Recently, some studies applied this model to deal with assessing healthcare utilisation in LMICs [11, 12]. According to a systematic review of 16 studies on the BM, the measurement of the concepts P, E, and N was found inconsistent and widely variable in the models depicted [31] given their specific context. However, no study that has adopted BM to explore the determinants of health-seeking behaviour for children under five with acute diarrhoea yet, especially in the DRC, such an undeveloped country has given there’s extensive evidence on the independent effects of rural-urban environment and wealth status on access to water and sanitation services [32]. Thus, we tended to evaluate healthcare utilisation, first aiming to report the status of healthcare utilisation for children under-five with diarrhoea. Moreover, structural equation modelling (SEM) techniques from the BM were used in this study to examine which factors were associated with healthcare utilisation for child diarrhoea in the DRC and to what extent they could impact. We expected our findings to contribute towards evidence on the determinants of healthcare utilisation, and to provide guidance for the development of interventions and policies with specific and comparative information in the DRC [33].

### Theoretical models and hypotheses

#### The Andersen behavioural model

**Fig. 1:**
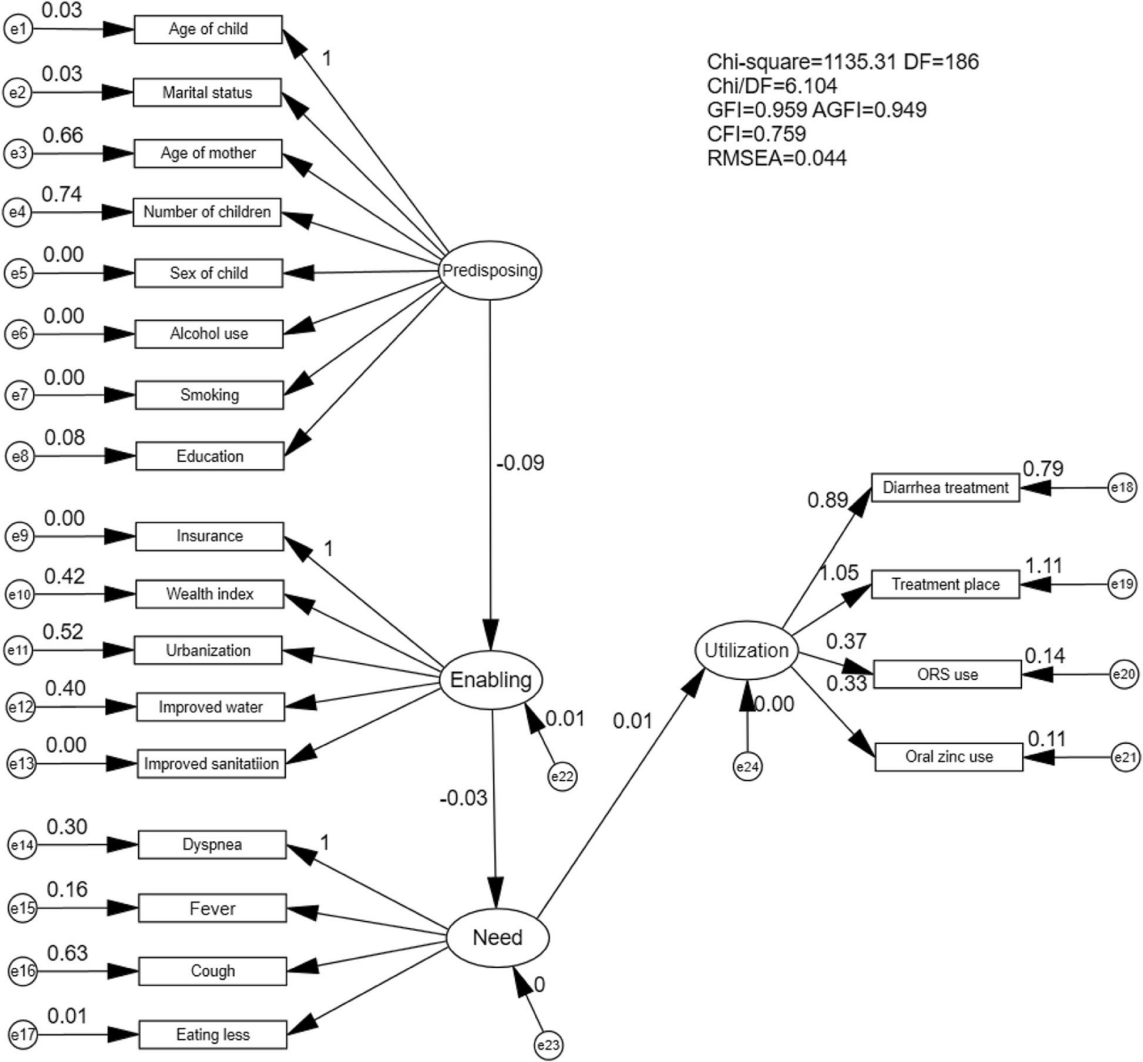
Empirically derived Structural Equation Model with standardized path coefficients

The most frequently cited model of health services use, Andersen’s Behavioural Model of Health Services Use [28] (BM), is widely accepted and used to study predictors of general health services use [22]. A systematic review showed that BM was explicitly employed as the theoretical background for a broad range of health services sectors and diseases [31]. The variants of Andersen’s model have proven exceedingly versatile and have been successfully used to explain health services use among children with diarrhoea [11, 12]. Although BM evolved over time, the modifications and additions did not change the fundamental components of the model, nor their relationships [34]. Various versions of the model suggest that health services use is a function of predisposing characteristics (including gender, age, and health beliefs), enabling resources that facilitate access to health services (such as wealth, social support, or community characteristics) and, most importantly, health need. Consistent with previous studies using BM, the following hypotheses are proposed to investigate the research questions (Fig. 1) [27, 31].

#### Predisposing characteristics

Predisposing characteristics (P) are “personal characteristics which exist prior to the onset of specific episodes of illness” [34]. These characteristics are primarily social and demographic factors, which may differ between individuals, and these factors impact the level of medical services utilisation. A systematic review of studies using BM identified the most frequently examined predisposing variables to be: age, marital status, sex, education level, ethnicity, and employment status [31].

Hypothesis 1 (H1): Predisposing characteristics positively influence patients’ use of health services even though the characteristics are not directly responsible for health services uses.

#### Enabling resources

Financing and organizational factors are considered to serve as criteria for enabling health services. The extent of family economic resources and the source of medical care, as well as the certain characteristics of the community in which the family lives all contribute to the health services use. Traditional enabling variables include health insurance, family income and the regular source of healthcare. Then, we expected to explore and predict more by finding out which is the most desirable form of the patients’ enabling resources in considering healthcare utilisation.

Hypothesis 2 (H2): Enabling resources positively influence patients’ use of healthcare services.

#### Health needs

Health needs are defined as the level of illness perceived by the patients’ caregivers [35]. Need represents the most immediate cause of health services use. Traditional need variables include the perception of poor health and specific health conditions. Previous studies often included morbidity and disability [34]. Once health needs are present, patients seek medical care, making health needs the most important component leading to healthcare use.

Hypothesis 3 (H3): Health needs have a strong influence on patients’ use of health utilisation.

## Methods

### Study design

This study used data from the Multiple Indicator Cluster Surveys (MICS) carried out in the DRC in 2018. The surveys were conducted by the National Statistical Office (NSO) and United Nations International Children’s Emergency Fund (UNICEF), in collaboration with relevant DRC Ministries. During data collection, following UNICEF guidelines, written informed consent was obtained from all participants (mothers) following an explanation of study objectives, assurance of the confidentiality of their identity, and a guarantee that there was no disadvantage to not participating in the study. All data were recruited anonymously via study identification numbers. A two-stage sampling method was used. At the first stage, three strata within each province, except Kinshasa, were created. Within each stratum, primary sample units (PSU) were selected with probability proportional to population size. In the second stage, 30 households were drawn from each of the 721 clusters, with 21,630 households selected in total. Details of these MICS have been previously described elsewhere [36]. For this study, MICS data were accessed and analyzed with the authorization of UNICEF.

The MICS were designed to collect updated information on the situation of women and children nationally. Due to the intrinsic structure of the questionnaire, only women who had children under 5 years old were included, a total of 20,245 participants. In our study, we focus on 2918 children who had diarrhoea in the last 2 weeks. Of that group, 292 participants were excluded due to missing values in maternal age, child age, sex of the child, marital status, maternal educational attainment, maternal smoking status, maternal alcohol use, wealth index, children’s health insurance, residence region, dyspnoea in the last 2 weeks, fever or cough in the last 2 weeks, seeking advice or treatment for diarrhoea in the last 2 weeks, Oral Rehydration Salts (ORS) or oral zine use, and type of services accessed for diarrhoea. The final analyses included a total of 2626 individuals.

### Measurement and data collection

Predisposing characteristics (P) are personal characteristics that exist prior to the onset of specific episodes of illness. In this study, P included age, sex, marital status, and maternal educational attainment as they have been previously used in studies examining risk factors for healthcare utilisation. Our study also included other factors such as children’s number in a household, smoking status, and alcohol use as they are often considered in studies using the BM. Age was a continuous variable, while the six others were categorical variables: sex (girl/boy), marital status (married/cohabiting/not married), maternal educational attainment (no formal school, primary school, junior high school, senior middle school, senior high school and above), children’s number in a household (1-13), maternal smoking status (yes/no), and maternal alcohol use (yes/no).

Enabling resources (E) are factors that make health services resources available to the family. In prior studies, the most commonly used variables in the category were income and financial situation. The current study attempted to capture E by examining five variables: wealth index (0-10), health insurance (yes/no), residence region (rural/urban), improved water sources (yes/no) and improved sanitation facilities (yes/no). The use of a wealth index is generally considered an accurate mechanism to determine socioeconomic status (SES) within a population [37, 38]. The wealth index [39], which captures underlying long-term wealth through household asset information, was constructed by principal components analysis using the information on ownership of consumer goods (e.g. refrigerators, televisions, cars, trucks, bicycles, and motorcycles), materials used in household construction (e.g. wood, bricks, rocks, and cement), household electricity, access to drinking water and water for general use, and improved sanitation facilities [36, 40]. Therefore, individuals’ SES was determined through the wealth index. Drinking water sources are defined as improved drinking water sources (piped water, boreholes or tubewells, protected dug wells, protected springs, rainwater, and packaged or delivered water), and unimproved sources (unprotected springs and wells, surface water, and other sources) [41]. Improved sanitation facilities are those designed to hygienically separate excreta from human contact (Flush/pour flush toilets connected to piped sewer systems, septic tanks or pit latrines; ventilated improved pit latrines, composting toilets or dry pit latrines with slabs), while unimproved sanitation facilities (Pit latrines without a slab or platform, hanging latrines or bucket latrines and open defecation). Improved and unimproved water sources or sanitation facilities were represented as dichotomous variables, with “1″ representing “unimproved” and “2″ representing “improved”, respectively for both water sources and sanitation (Additional file 1: Table 1) [32].

We measured children’s health needs (N) by using the following common factors that impacted the possibility of healthcare-seeking behaviour, including fever (yes/no), cough (yes/no), dyspnoea (yes/no), and eating less (No, eating more/Same as before/Yes, eating less) in the last 2 weeks.

Four variables were used in this study to quantify children’s healthcare usage for diarrhoea: seeking advice or treatments for diarrhoea in the last 2 weeks (yes/no), the type of services accessed for diarrhoeal health issues (including 16 items, the options given for “where did you seek advice or treatment?” were classified as the public health sector, private health sector, other sources, and none (Additional file 1: Table 1) [42], ORS use in the last 2 weeks (yes/no), and oral zinc use in the last 2 weeks (yes/no).

### Data analysis

Structural equation modelling (SEM) was conducted using SPSS AMOS, version 24 (IBM-SPSS, Chicago), to validate the hypothesized direct and indirect associations among individual characteristics, health services quality contextual factors, and healthcare-seeking behaviours. To determine whether the relationships among the latent variables constructed were as suggested by BM, a confirmatory factor analysis (CFA) was conducted. Guided by the BM, four latent variables were examined: predisposing characteristics, enabling characteristics, health needs, and health services use for children with diarrhoea.

In addition to the chi-squared test (χ^2^), the goodness of fit index (GFI), the adjusted goodness of fit index (AGFI), and the comparative fit index (CFI) were examined. In all cases, the values ranged from 0 to 1 and reflected the improvement in the fit of a hypothesized model over a model of independence among the measured variables, with values over 0.95 indicative of a good fit. Eventually, the root means squared error of approximation (RMSEA) was used as a measure of model fitness per degrees of freedom, with values less than 0.06 considered desirable.

## Results

### Descriptive findings

The percentage, frequency, mean value, and variance for each variable in the P, E and N factors used in the model are presented in Table 1. In the final analysis, of the 2626 participants included, 51.4% were boys, and the mean age was 1.61 years (SD = 1.28). Only about one-third of children under-five with diarrhoea (34.1%) had access to improved water sources, and 44.8% of children had access to improved sanitation facilities. Over 2 weeks before the survey, the proportion of participants who had fever, cough, dyspnoea, or eating less were 54.8, 40.9, 17.4, and 68.20% respectively. Amongst children under-five with diarrhoea, more than half of them (52.0%) did not receive any healthcare. Nearly one in five of children under-five with diarrhoea (19.7%) sought care in the public health sector, and a quarter of them sought care in the private health sector or at home.

**Table 1:** Summary statistics of measured variables in the Andersen Behavioural Model for healthcare utilisation (*n* = 2626)

Variables	Mean/N	SD/%
***Predisposing1***		
**Age of mother**	29.65	7.08
**Age of child**	1.61	1.28
**Number of children**	3.89	2.13
**Sex of Child**		
Girl	1277	48.63
Boy	1349	51.37
**Marital status**		
Unmarried	297	11.31
Cohabiting	396	15.08
Married	1933	73.61
**Maternal education attainment**		
No formal school	612	23.31
Primary school	1152	43.87
Middle school	370	14.09
Secondary school	463	17.63
High school and above	29	1.10
**Maternal smoking status**		
No	2568	97.79
Yes	58	2.21
**Maternal alcohol use**		
No	1610	61.31
Yes	1016	38.69
***Enabling***		
**Wealth index**	4.02	2.44
**Insurance**		
No	2571	97.91
Yes	55	2.09
**Region**		
Rural	1943	73.99
Urban	683	26.01
**Improved water sources**		
No	1730	65.88
Yes	896	34.12
**Improved sanitation facilities**		
No	1450	55.22
Yes	1176	44.78
***Need-for-Care***		
**Had a fever in the last two weeks**		
No	1187	45.20
Yes	1439	54.80
**Coughing in the last two weeks**		
No	1551	59.06
Yes	1075	40.94
**Dyspnoea in the last two weeks**		
No	2170	82.64
Yes	456	17.36
**Eating less in the last two weeks**		
No, eating more	106	4.04
Same as before	729	27.76
Yes, eating less	1791	68.20
***Utilization***		
**Seeking advice or treatment for diarrhoea**		
No	1366	52.02
Yes	1260	47.98
**ORS use**		
No	2021	76.96
Yes	605	23.04
**Oral zinc use**		
No	2153	81.99
Yes	473	18.01
**Place to seek care of diarrhoea**		
No	1366	52.02
Other sources	28	1.07
Family/friends	277	10.55
Private health sector	438	16.68
Public health sector	517	19.69

*ORS* Oral Rehydration Salts

### Measurement model

In the hypothesis model, this study had three latent variables: P, E, and N, all of which were exogenous variables. A separate measurement model was developed for each of the latent variables and then independently validated by CFA.

As previously mentioned, the predisposing characteristics variable (P) was composed of eight indicators: child age, sex of the child, maternal age, marital status, maternal educational attainment, children’s number in a household, maternal smoking status, and alcohol use. As depicted in Fig. 1, all indicators had a strong positive correlation with the latent variable P except for the sex of the child, smoking status and alcohol use, where the correlation was very weak. Also, all indicators for the construct were statistically significant at *p* < 0.05 except for the sex of the child and smoking status (Table 2, *p* = 0.435 and *p* = 0.599, respectively). Therefore, the sex of the child, smoking status and alcohol use were excluded from the modified/nested model. Refer to the modification suggests (Additional file 1: Table 2) and as well as considering the practical significance, maternal educational attainment was moved from P to E (Modification Indices = 145.489), urbanization was removed from the enabling construct and coughing was removed from the health need construct.

**Table 2:** Direct effects for the empirically derived Model A

Indicators		Latent variable	Standard β	β	S.E.	***p***-value
**Enabling**	<---	**Predisposing**	−0.094	−0.002	0.002	0.225
**Need**	<---	**Enabling**	−0.027	−1.030	1.404	0.463
**Utilization**	<---	**Need**	0.013	0.027	0.051	0.596
Age of mother	<---	Predisposing	0.810	25.634	3.258	< 0.001
Marital status	<---	Predisposing	0.184	0.545	0.110	< 0.001
Education	<---	Predisposing	0.290	1.140	0.200	< 0.001
Number of children	<---	Predisposing	0.860	8.193	1.262	< 0.001
Sex of child	<---	Predisposing	−0.017	−0.039	0.049	0.435
Alcohol use	<---	Predisposing	0.056	0.118	0.051	0.020
Smoking	<---	Predisposing	0.012	0.008	0.015	0.599
Age of child	<---	Predisposing	0.175	1.000		
Wealth index	<---	Enabling	0.650	239.565	160.392	0.135
Urbanization	<---	Enabling	0.721	50.777	34.348	0.139
Improved water sources	<---	Enabling	0.630	51.088	34.539	0.139
Improved sanitation facilities	<---	Enabling	0.022	1.961	2.610	0.452
Insurance	<---	Enabling	0.039	1.000		
Had a fever	<---	Need	0.400	0.954	0.067	< 0.001
Coughing	<---	Need	0.791	1.862	0.169	< 0.001
Eating less	<---	Need	0.079	0.210	0.064	0.001
Dyspnea	<---	Need	0.550	1.000		
Treatment place	<---	Utilization	1.052	3.943	0.075	< 0.001
ORS use	<---	Utilization	0.372	0.335	0.018	< 0.001
Oral zinc use	<---	Utilization	0.332	0.276	0.017	< 0.001
Seeking advice or treatment	<---	Utilization	0.891	1.000		

*ORS* Oral Rehydration Salts

The final enabling construct was composed of 5 indicators: maternal educational attainment, wealth index, health insurance, improved water source and improved sanitation facilities. The health need construct was composed of three indicators: the presence of fever, dyspnoea or eating less. As shown, all indicators had a strong positive correlation with the latent variable and were statistically significant (Figs. 3, 4).

### Structural model

**Fig. 2:**
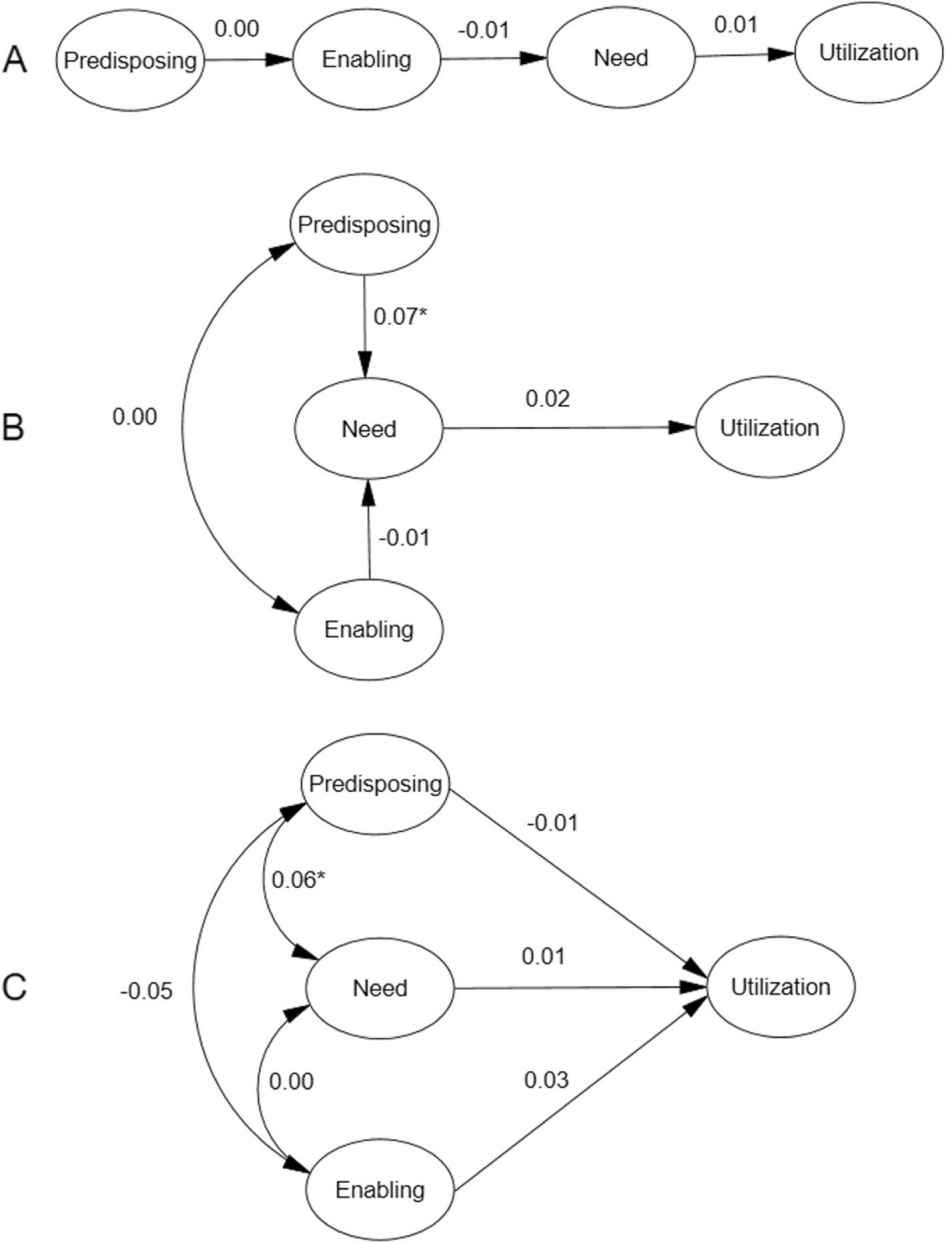
Empirically derived Structural Equation Model. *p < 0.05

**Fig. 3:**
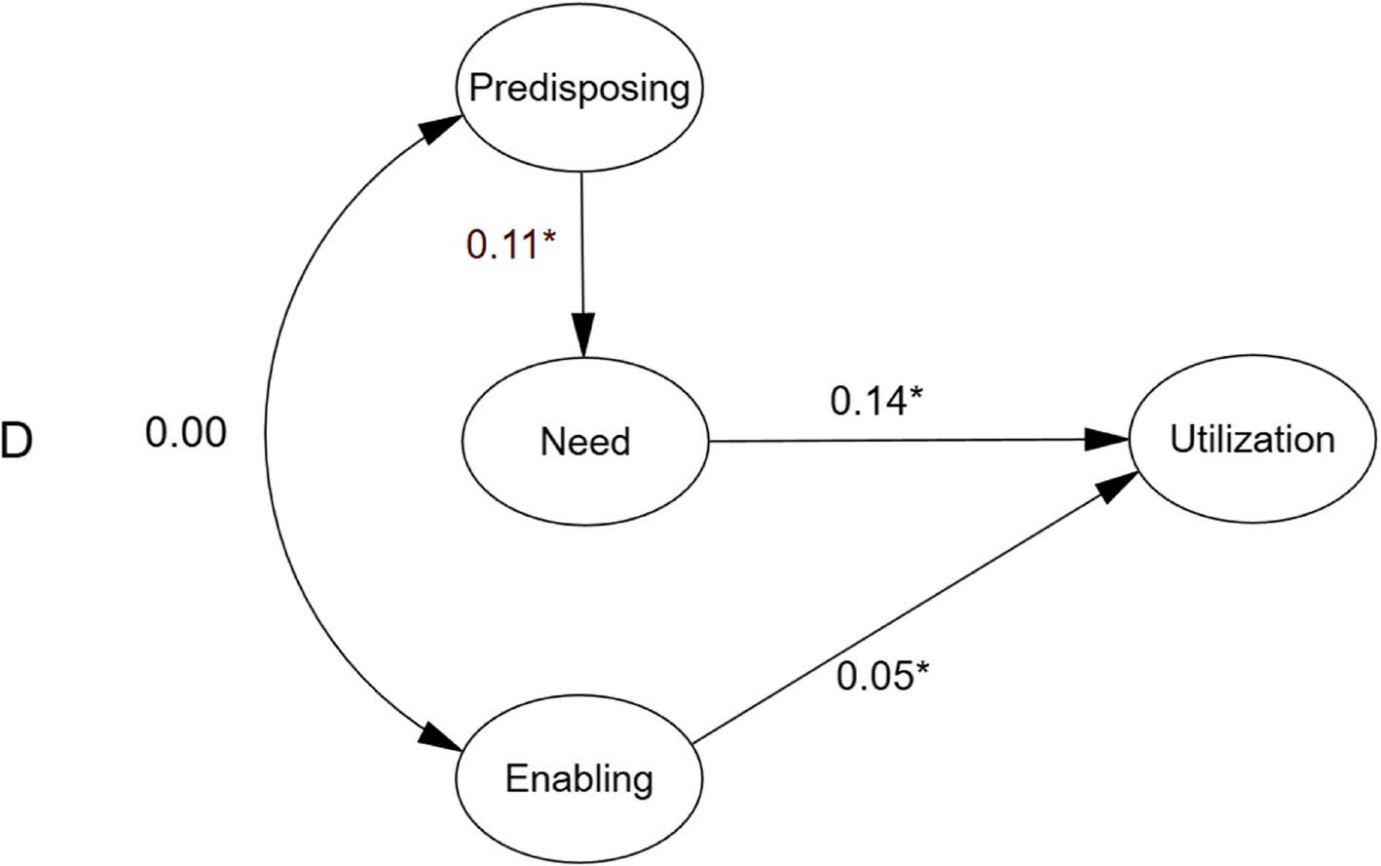
Modified Structural Equation Model. *p < 0.05

**Fig. 4:**
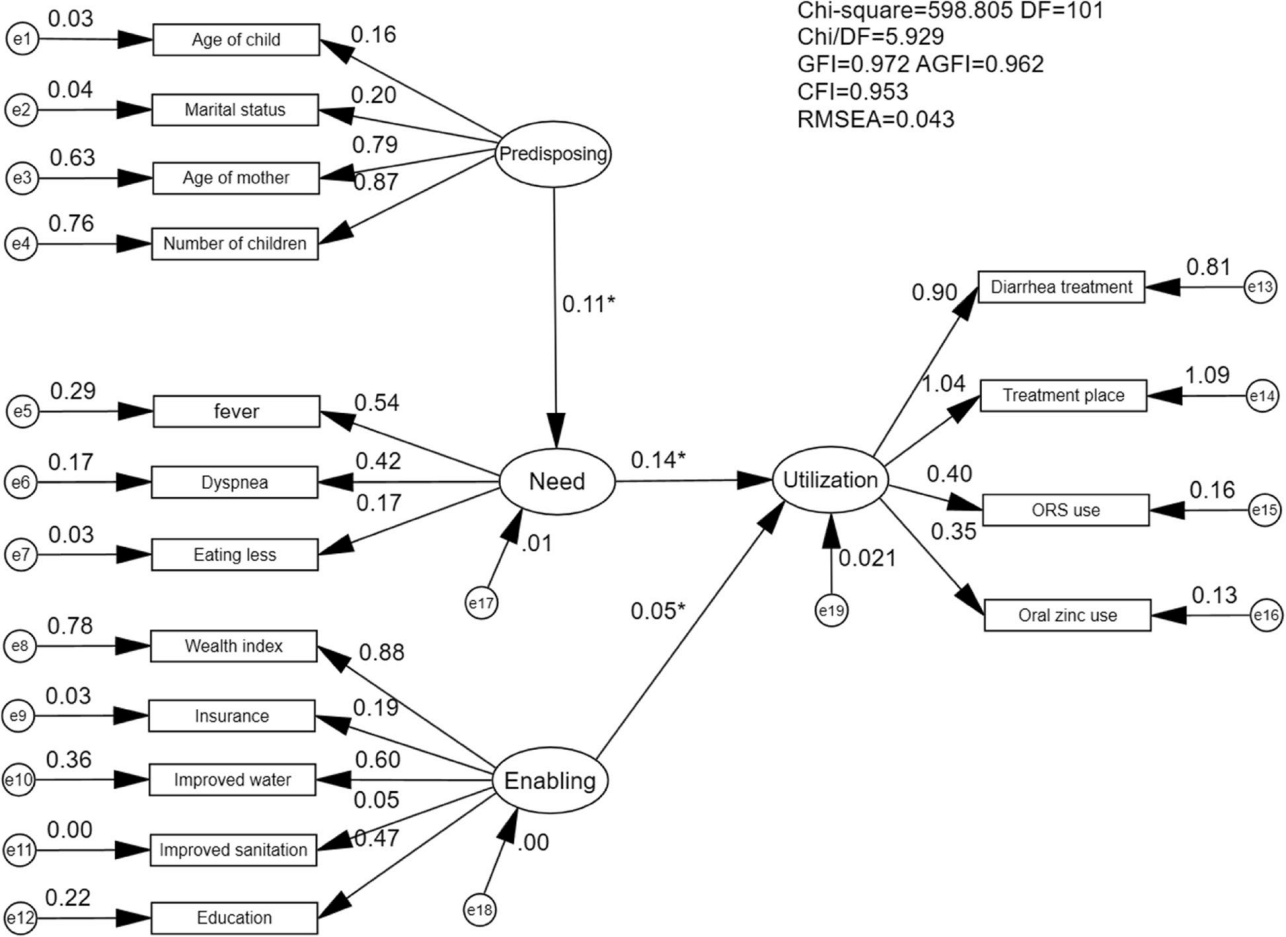
Final structural equation models with standardized path coefficients

After revising the measurement models for P, E, and N, three empirically derived models for health services uses for childhood diarrhoea were constructed based on the different versions of BM. Statistically significant relationships are depicted in Fig. 2. As shown in Fig. 2, considering hypothesis model A and hypothesis model B, E had a slight, non-significant influence on N (− 0.01, *p* = 0.85 and − 0.01, *p* = 0.76, respectively). In hypothesis model C, P had a slight, non-significant influence on healthcare utilisation (− 0.01, *p* = 0.52). None of the three hypothesis models has a good CFI, which indicates the model fitting results are not so satisfying. Applying Andersen’s model to the context of the DRC, minimal model modification was conducted, and the final model was acceptable, shown in Fig. 3 and Table 3, with a GFI of 0.972, CFI of 0.953 and RMSEA of 0.043 (90% CI: 0.040, 0.047). All factor loadings were significant (*p* < 0.001), indicating that the model was plausible. Figure 4 provides the standardized path coefficients. The model revealed the direct effects of latent variables. Among all the variables, “Need” had the largest direct effect on healthcare utilisation (β, direct effect = 0.135, *p* < 0.001), followed by “enabling resources” (β, direct effect = 0.051, *p* = 0.015). Indirect effects were also examined based on Andersen’s model. “Need” emerged as a mediator of the effect of “Predisposing” on “Utilisation” (β, indirect effect = 0.014; *p* = 0.009). All of the proposed relationships were positively associated with health services use. For example, higher enabling resources (especially household wealth [β = 0.88, *p* < 0.001]) and improved water sources [β = 0.60, *p* < 0.001]) and more health needs (especially fever [β = 0.54, *p* < 0.001]) were both positively associated with a greater chance of health services use. In this sample, greater predisposing characteristics (especially the number of children [β = 0.87, *p* < 0.001]) were associated with a higher likelihood of healthcare utilisation (Fig. 4).

**Table 3:** Fit indices for the measurement and structural models

Model	X^**2**^/df	***P***-value	RMSEA	(95%CI)	GFI	AGFI	CFI	Criteria fitted
Hypothesis model A	5.299	< 0.001	0.04	0.037-0.044	0.972	0.964	0.863	3
Hypothesis model B	5.295	< 0.001	0.04	0.037-0.044	0.973	0.964	0.864	3
Hypothesis model C	5.981	< 0.001	0.044	0.040-0.047	0.972	0.961	0.869	3
Modified model	5.929	< 0.001	0.043	0.040-0.047	0.972	0.962	0.953	4

*χ*^*2*^ chi-squared test, *RMSEA* the root means squared error of approximation, *GFI* the goodness of fit index, *AGFI* the adjusted goodness of fit index, *CFI* the comparative fit index

## Discussion

Using Andersen’s Behavioural Model of Health Services Use, healthcare utilisation and predictors of healthcare-seeking behaviour among children under-five years old with diarrhoea in the DRC were examined. The results indicate that in the DRC, greater than half of the children that reported having diarrhoea did not receive any treatment. Out of those who sought care, utilisation of government health facilities was preferred, but nearly one quarter sought healthcare services either at home or in the private health sector.

In previous studies, all latent variables were either directly linked to the main outcome, “healthcare utilisation”, or had an orderly monoline influence that subsequently impacted the healthcare utilisation. However, this study found that in the context of the DRC, a new modified version of the model combining both methods of demonstrating relationships between the two variables may more accurately describe health services use by children under under-five with diarrhoea than the former model utilised in past studies in high-income countries. This modified model was built around four latent variables, which corresponded with healthcare services use described by Andersen [31]. Also, this analysis suggested that health needs were a key factor in predicting the use of health services when treating diarrhoea amongst children under-five in the DRC. Enabling alternative access was directly linked to a higher likelihood of using services. Predisposing conditions only affected the use of health services through the needed resources variable.

According to Andersen’s model, enabling factors are indicators of whether children will use the needed health services to treat disease. Generally speaking, we found that access to improved water and sanitation, as well as SES factors like household wealth, were significantly associated with healthcare-seeking behaviours for diarrhoea treatment in the DRC. The largest declines in risk exposure from 2010 to 2019 are strongly linked to social and economic development, including unsafe water, sanitation, and handwashing [43]. A systematic analysis for the Global Burden of Disease (GBD) Study shows that unimproved water sources and unimproved sanitation facilities were the leading risk factors for diarrhoea, responsible for 72 and 56% of diarrhoea deaths in children younger than five years, respectively [44]. The MDGs set a target that by 2015, 88% of the population had access to improved water sources, and 75% had access to improved sanitation. The reality is that endeavour is still needed in DRC. Marginalized populations always suffer the combined effects of unimproved water sources and poor sanitation [45], consistent with the previous evidence, our study shows the necessity to reduce the inequalities in the coverage of access to improved water sources and sanitation in DRC. In addition, governments should strengthen and ensure the construction of improved water sources and sanitation facilities in the vicinity of households.

Low SES was a major deterrent for mothers seeking healthcare in many developing countries [46]. A study in India showed a clear gradient across wealth quintiles with richer and richest groups having the higher and highest likelihoods for seeking treatment from healthcare providers [11]. In the DRC, the healthcare system is a fee-for-service program financed almost entirely by user fees and external donor money [47]. Minimal government financing at the medical facilities level is provided, so accessing healthcare for one’s children is a financial burden for individuals of lower SES [48].

This study reported a positive association between the level of maternal education and appropriate healthcare-seeking behaviours, which was consistent with another systematic review conducted in developing countries [49]. We found that mothers with lower educational attainment were less likely to seek care [12]. Similar findings from Sub-Saharan Africa indicate that promoting enrolment in secondary education or above would be valuable to the health of under-five children [50].

People with diarrhoea caused by some infections may have common symptoms, including fever (35%) [51]. It has been reported that fever is the most frequent reason for caretakers seeking treatment for their under-five children [52]. Diarrhoea is not fatal itself, the delay and inappropriately healthcare utilisation leads to a high degree of mismanagement and resultant severe and life-threatening complications [53]. The low-cost and highly effective solution to reduce child mortality from diarrhoea is ORS therapy. Yet today only about 23% of children with diarrhoea in the DRC receive ORS therapy. These low levels highlight the urgent need to educate caregivers on appropriate treatment [9]. In Africa, healthcare utilisation for diarrhoea in children is still low and considerable efforts are required to educate parents about diarrhoea and its prevention and proper treatment [53].

### Strengths and limitations

One of the strengths of screening a national sample is that the standardized methodology used by MICS allows for comparisons with similar data from other countries. This permitted horizontal comparisons to be made by exploring different influential factors on health services. Additionally, a conceptual framework was applied based on Andersen’s health behavioural model, a widely accepted method for assessing factors associated with healthcare utilisation. This study then developed modified models to predict healthcare utilisation for diarrhoea in children under five years in the DRC.

As most of the examination was based on Anderson’s behavioural model, which posited a particular directionality of influence in health services use, BM was also confirmed by using a secondary dataset [31]. However, as cross-sectional data were used, the nature of any causal relationships among these three latent variables (predisposing characteristics, enabling factors, and need factors) and healthcare utilisation for children under five with diarrhoea in the DRC was unable to be ascertained. Furthermore, only one dataset was used to test different versions of Andersen’s model (traditional and empirically modified versions), and then to explore the relationships among the various latent factors. This work is expected to soon be replicated with different datasets from other developing countries to examine health services use in a variety of contexts.

Although the BM was explicitly employed as the theoretical background for the reviewed studies, there was a lack of consistency in these findings. As previously mentioned, the majority of reviewed studies’ operationalizations of the model revealed that only a small set of variables were commonly used and that there were many variations in the way these variables were categorized, especially concerning predisposing and enabling factors. This may stem from the secondary datasets used in the majority of the studies, which limited the variables available for study. Therefore, more primary studies exploring healthcare utilisation in the DRC or similar contexts are urgently needed to enrich understanding of this subject.

## Conclusion

To promote the use of health services among children under-five years to treat diarrhoea, we need to comprehend what encourages and discourages the use of such services. Evidence was found suggesting that a variant of Andersen’s model successfully depicts healthcare utilisation in the DRC for children under-five years with diarrhoea. Similar to other studies using the 1995 version of the model, the BM used in this study could be used to reflect the influences on health services use for children’s diarrhoea and health status by including feedback loops representing the influences of the outcome, predisposing factors, enabling factors, and perceived need [18].

Foremost, improvements in access to safe water and adequate sanitation are required to make children healthier and less likely to develop infections that lead to diarrhoea. Meanwhile, oral rehydration therapy coupled with educating caregivers on its appropriate use is needed. Furthermore, health education to caregivers about diarrhoea, its causes prevention and proper treatment among children under-five is an important factor for initiating healthcare utilisation. After getting aware of the needs for a health issue, the E is directly linked to people’s healthcare utilisation since affordability and access to healthcare is crucial when seeking help.

## Supplementary Information

**Additional file 1.**

## Data Availability

This study used data from the Multiple Indicator Cluster Surveys (MICS-Palu) carried out in DRC, a nationally representative household survey of children aged from 0 to 5, females aged from 15 to 49, and males aged from 15 to 59 (https://mics.unicef.org/surveys).

## Abbreviations

MICS: Multiple Indicator Cluster Surveys
DHS: Demographic and Health Survey
DRC: Democratic Republic of the Congo
SSA: Sub-Saharan Africa
WHO: World Health Organization
UNICEF: United Nations International Children’s Emergency Fund
MDGs: Millennium Development Goals
SDG: Sustainable Development Goal
SEM: Structural Equation Modelling
CFA: Confirmatory Factor Analysis
GFI: Goodness of Fit Index
CFI: Comparative Fit Index
AGFI: AdjustedGoodness of Fit Index
RMSEA: Root Means Squared Error of Approximation
SD: Standard Deviation
LMICs: Low- and Middle-Income Countries
BPL: Below Poverty Line
BM: Behavioural Model
P: Predisposing characteristics
E: Enabling resources
N: Needs
NSO: National Statistical Office
PSU: Primary Sample Units
ORS: Oral Rehydration Salts
SES: Socioeconomic status
SEP: Socioeconomic position

## References

[1] Wardlaw T, Salama P, Brocklehurst C, Chopra M, Mason E (2010). Diarrhoea: why children are still dying and what can be done. Lancet.

[2] Millennium Development Goals (MDGs) [https://www.who.int/news-room/fact-sheets/detail/millennium-development-goals-(mdgs)].

[3] World Health O (2015). Improving health system efficiency: Democratic Republic of the Congo: improving aid coordination in the health sector.

[4] Butcher F, Galanek JD, Kretschmar JM, Flannery DJ (1982). The impact of neighborhood disorganization on neighborhood exposure to violence, trauma symptoms, and social relationships among at-risk youth. Soc Sci Med.

[5] WHO/maternal and infant mortality [https://www.who.int/news-room/fact-sheets/detail/maternal-mortality].

[6] UNICEF/WHO (2019). Progress on household drinking water, sanitation and hygiene 2000-2017. Special focus on inequalities.

[7] Democratic Republic of the Congo, Key demographic indicators [https://data.unicef.org/country/cod/].

[8] Initiative EC (2020). CHILD HEALTH IN DRC.

[9] UNICEF/WHO (2009). Diarrhoea: why children are still dying and what can be done.

[10] Podewils LJ, Mintz ED, Nataro JP, Parashar UD (2004). Acute, infectious diarrhea among children in developing countries. Semin Pediatr Infect Dis.

[11] Sreeramareddy CT, Sathyanarayana TN, Kumar HN (2012). Utilization of health care services for childhood morbidity and associated factors in India: a national cross-sectional household survey. PLoS One.

[12] Adane M, Mengistie B, Mulat W, Kloos H, Medhin G (2017). Utilization of health facilities and predictors of health-seeking behavior for under-five children with acute diarrhea in slums of Addis Ababa, Ethiopia: a community-based cross-sectional study. J Health Popul Nutr.

[13] Aday LA, Andersen R (1974). A framework for the study of access to medical care. Health Serv Res.

[14] Ricketts TC, Goldsmith LJ (2005). Access in health services research: the battle of the frameworks. Nurs Outlook.

[15] Murray CJL, Evans DB, Murray CJL, Evans DB, World Health Organization (2003). Global Programme on Evidence for Health P: Health systems performance assessment : debates, methods and empiricism.

[16] Levesque JF, Harris MF, Russell G (2013). Patient-centred access to health care: conceptualising access at the interface of health systems and populations. Int J Equity Health.

[17] Penchansky R, Thomas JW (1981). The concept of access: definition and relationship to consumer satisfaction. Med Care.

[18] Andersen RM. Revisiting the behavioral model and access to medical care: does it matter? J Health Soc Behav. 1995;36(1):1-10. 7738325

[19] Andersen RM, Rice TH, Kominski GF. Changing the U.S. health care system: Key issues in health services policy and management (3rd ed). San Francisco: Jossey-Bass; 2007.

[20] Bradley EH, McGraw SA, Curry L, Buckser A, King KL, Kasl SV, Andersen R (2002). Expanding the Andersen model: the role of psychosocial factors in long-term care use. Health Serv Res.

[21] Andersen RM. Families' use of health services: a behavioral model of predisposing, enabling and need components [dissertation] West Lafayette. In: Purdue University; 1968.

[22] Andersen R, Newman JF. Societal and individual determinants of medical care utilization in the United States. Milbank Mem Fund Q Health Soc. 1973:95–124. 4198894

[23] Holde GE, Baker SR, Jönsson B (2018). Periodontitis and quality of life: what is the role of socioeconomic status, sense of coherence, dental service use and oral health practices? An exploratory theory-guided analysis on a Norwegian population. J Clin Periodontol.

[24] Andersen R, Davidson P (1997). Ethnicity, aging, and oral health outcomes: a conceptual framework. Adv Dent Res.

[25] Muirhead V, Quinonez C, Figueiredo R, Locker D (2009). Predictors of dental care utilization among working poor Canadians. Community Dent Oral Epidemiol.

[26] Baker S (2009). Applying Andersen’s behavioural model to oral health: what are the contextual factors shaping perceived oral health outcomes?. Community Dent Oral Epidemiol.

[27] Graham A, Hasking P, Brooker J, Clarke D, Meadows G (2017). Mental health service use among those with depression: an exploration using Andersen's behavioral model of health service use. J Affect Disord.

[28] Andersen R. A behavioral model of families’ use of health services. Chicago: Center for Health Administration Studies, University of Chicago; 1968. Research Series No. 25:16-121.

[29] Andersen RM, Rice TH, Kominski GF. Changing the U.S. health care system: Key issues in health services policy and management. Jossey-Bass. 2001;2:1-454.

[30] Azage M, Kumie A, Worku A, Bagtzoglou AC (2016). Childhood diarrhea in high and low hotspot districts of Amhara region, Northwest Ethiopia: a multilevel modeling. J Health Popul Nutr.

[31] Babitsch B, Gohl D, von Lengerke T (2012). Re-revisiting Andersen's behavioral model of health services use: a systematic review of studies from 1998-2011. Psychosoc Med.

[32] Armah FA, Ekumah B, Yawson DO, Odoi JO, Afitiri AR, Nyieku FE (2018). Access to improved water and sanitation in sub-Saharan Africa in a quarter century. Heliyon.

[33] Fosu GB (1994). Childhood morbidity and health services utilization: cross-national comparisons of user-related factors from DHS data. Soc Sci Med.

[34] Andersen RM (2008). National health surveys and the behavioral model of health services use. Med Care.

[35] Wright J, Williams R, Wilkinson JR (1998). Development and importance of health needs assessment. BMJ.

[36] National Statistical Institute (Congo, DR), Ministry of Planning (Congo, DR), United Nations Children's Fund (UNICEF). Congo, DR Multiple Indicator Cluster Survey 2010. New York: United Nations Children's Fund (UNICEF); 2011.

[37] Vyas S, Kumaranayake L (2006). Constructing socio-economic status indices: how to use principal components analysis. Health Policy Plan.

[38] Gwatkin DR, Rutstein S, Johnson K, Suliman E, Wagstaff A, Amouzou A (2007). Socio-economic differences in health, nutrition, and population within developing countries: an overview. Niger J Clin Pract.

[39] Howe LD, Hargreaves JR, Gabrysch S, Huttly SR (2009). Is the wealth index a proxy for consumption expenditure? A systematic review. J Epidemiol Community Health.

[40] Rutstein SO (2015). Steps to constructing the new DHS wealth index.

[41] WHO, UNICEF (2019). WASH in health care facilities: Global Baseline Report 2019.

[42] Carter ED, Ndhlovu M, Munos M, Nkhama E, Katz J, Eisele TP (2018). Validity of maternal report of care-seeking for childhood illness. J Glob Health.

[43] Collaborators GBDRF (2020). Global burden of 87 risk factors in 204 countries and territories, 1990-2019: a systematic analysis for the global burden of disease study 2019. Lancet.

[44] Collaborators GBDDD (2018). Estimates of the global, regional, and national morbidity, mortality, and aetiologies of diarrhoea in 195 countries: a systematic analysis for the global burden of disease study 2016. Lancet Infect Dis.

[45] Pullan RL, Freeman MC, Gething PW, Brooker SJ (2014). Geographical inequalities in use of improved drinking water supply and sanitation across sub-Saharan Africa: mapping and spatial analysis of cross-sectional survey data. PLoS Med.

[46] Ahmed S, Creanga AA, Gillespie DG, Tsui AO (2010). Economic status, education and empowerment: implications for maternal health service utilization in developing countries. PLoS One.

[47] Dumbaugh M, Bapolisi W, van de Weerd J, Zabiti M, Mommers P, Balaluka GB, Merten S (2017). Evaluating the comparative effectiveness of different demand side interventions to increase maternal health service utilization and practice of birth spacing in south Kivu, Democratic Republic of Congo: an innovative, mixed methods approach. BMC Pregnancy Childbirth.

[48] Fox S, Witter S, Wylde E, Mafuta E, Lievens T (2014). Paying health workers for performance in a fragmented, fragile state: reflections from Katanga Province, Democratic Republic of Congo. Health Policy Plan.

[49] Geldsetzer P, Williams TC, Kirolos A, Mitchell S, Ratcliffe LA, Kohli-Lynch MK, Bischoff EJ, Cameron S, Campbell H (2014). The recognition of and care seeking behaviour for childhood illness in developing countries: a systematic review. PLoS One.

[50] Kassile T, Mmbando BP, Lokina R, Mujinja P (2018). Perceptions of caretakers with different socioeconomic status about the harmful outcomes of fever in under-five children in Dodoma region, Central Tanzania: a cross-sectional study. Alex J Med.

[51] Abu-Elyazeed RR, Wierzba TF, Frenck RW, Putnam SD, Rao MR, Savarino SJ, Kamal KA, Peruski LF, Messih IAAE, El-Alkamy SA (2004). Epidemiology of Shigella-associated diarrhea in rural Egyptian children. Am J Trop Med Hyg.

[52] Ravanipour M, Akaberian S, Hatami G (2014). Mothers' perceptions of fever in children. J Educ Health Promot.

[53] Fikire A, Ayele G, Haftu D. Determinants of delay in care seeking for diarrheal diseases among mothers/caregivers with under-five children in public health facilities of Arba Minch town, southern Ethiopia; 2019. PLoS One. 2020;15(2):e0228558. 10.1371/journal.pone.0228558 PMC7018063 32053615

