# Supplementary material for: Understanding the context of healthcare utilisation for children under-five with diarrhoea in the DRC: based on Andersen behavioural model
Source: BMC Health Serv Res. 2022 Feb 4;22:144. doi: 10.1186/s12913-022-07530-4 (PMC8815172; doi:10.1186/s12913-022-07530-4)
Supplement: Supplementary file 1 — Additional file 1. [file 12913_2022_7530_MOESM1_ESM.docx]

**Additional file 1 Table 1 The concepts and constructs of Andersen’s behavioural models.**

| **Latent Variables** | **Indicators** |  | **Description** |
| --- | --- | --- | --- |
| **Predisposing** |  |  |  |
| Age of mother | Age of mother |  | Continuous variable: 15-49 years |
| Age of child | Age of child |  | Continuous variable: 0-4 years |
| Marital status | Marital status |  | 1=Unmarried;2=Cohabiting; 3=Married |
| Number of children | Number of children |  | Continuous variable: 0-13 |
| Sex of child | Sex of child |  | 1=Girl; 2=Boy |
| Maternal tobacco use | Maternal tobacco use |  | 1=No; 2=Yes |
| Maternal alcohol use | Maternal alcohol use |  | 1=No; 2=Yes |
| Maternal educational attainment | Maternal educational achievement |  | 5 level (1 no formal school, 2 primary school, 3 junior high school, 4 senior middle school, 5 senior high school and above) |
| **Enabling** |  |  |  |
| Wealth index | Wealth index |  | 10 quantile |
| Insurance | Insurance |  | 1=No; 2=Yes |
| Region | Urbanization |  | 1=Rural; 2=Urban |
| Drinking water sources | Improved water sources |  | 1=No (Unprotected dug well, unprotected spring, river, dam, lake, pond, stream, canal and irrigation canal);  2=Yes (Piped water, boreholes or tubewells, protected dug wells, protected springs, rainwater, and packaged or delivered water) |
| Sanitation facilities | Improved sanitation facilities |  | 1=No (Pit latrines without a slab or platform, hanging latrines or bucket latrines and open defecation);  2=Yes (Flush/pour flush to piped sewer systems, septic tanks or pit latrines; ventilated improved pit latrines, composting toilets or pit latrines with slabs) |
| **Need-for-Care** |  |  |  |
| Had a fever | Had a fever in the last two weeks |  | 1=No; 2=Yes |
| Coughing | Coughing in the last two weeks |  | 1=No; 2=Yes |
| Dyspnoea | Difficulty in breathing in the last two weeks |  | 1=No; 2=Yes |
| Eating less | Eating less in the last two weeks |  |  |
| **Utilization** |  |  |  |
| Seeking advice or treatment | Seeking advice or treatment for diarrhoea |  | 1=No; 2=Yes |
| ORS use | ORS use |  | 1=No; 2=Yes |
| Oral zinc use | Oral zinc use |  | 1=No; 2=Yes |
| Place to seek care | Place to seek care of diarrhoea |  | 16 items, classified into 4 categories: 1 none;  2 other sources (Relative/Friend, Shop/Market/Street, Traditional Practitioner);  3 private health sector (Private hospital/clinic, Pharmacy, Private doctor, Mobile hospital/clinic, Community based agent/fieldworker, Other private sectors);  4 public health sector (Government hospital, Government health centre, Mobile hospital/clinic, Community based agent/fieldworker, Other public sectors) |

**Additional file 1 Table 2 Suggestions for modification about Model A**

| **Indicators** |  | **Latent variable** | **Modification Indices** | **Par Change** |
| --- | --- | --- | --- | --- |
| Maternal educational attainment | <--- | Enabling | 145.489 | -48.098 |
| Improved sanitation facilities | <--- | Need | 7.038 | 0.15 |
| Improved sanitation facilities | <--- | Utilization | 12.046 | -0.071 |
| Improved water sources | <--- | Need | 4.494 | 0.093 |
| smoking | <--- | Need | 6.81 | 0.044 |
| Alcohol consumption | <--- | Need | 14.365 | 0.208 |
| Coughing | <--- | Utilization | 11.112 | -0.059 |
| Urbanization | <--- | Predisposing | 12.422 | -0.111 |
| Urbanization | <--- | Utilization | 11.493 | -0.046 |

ORS: Oral Rehydration Salts

**Additional file 1 Table 3 Direct effects for the final model**

| **Indicators** |  | **Latent variable** | **Standard β** | **β** | **S.E.** | **p-value** |
| --- | --- | --- | --- | --- | --- | --- |
| Need | <--- | Predisposing | 0.105 | 0.134 | 0.051 | 0.009 |
| Utilization | <--- | Need | 0.135 | 0.227 | 0.058 | <0.001 |
| Utilization | <--- | Enabling | 0.051 | 0.011 | 0.004 | 0.015 |
| Age of mother | <--- | Predisposing | 0.791 | 26.548 | 3.667 | <0.001 |
| Marital status | <--- | Predisposing | 0.201 | 0.647 | 0.139 | <0.001 |
| Number of children | <--- | Predisposing | 0.870 | 8.801 | 1.673 | <0.001 |
| Age of child | <--- | Predisposing | 0.164 | 1.000 |  |  |
| Had a fever | <--- | Need | 0.539 | 1.000 |  |  |
| Dyspnoea | <--- | Need | 0.417 | 0.588 | 0.132 | <0.001 |
| Eating less | <--- | Need | 0.174 | 0.362 | 0.097 | <0.001 |
| Wealth index | <--- | Enabling | 0.881 | 1 |  |  |
| Education | <--- | Enabling | 0.473 | 0.23 | 0.014 | <0.001 |
| Insurance | <--- | Enabling | 0.186 | 0.012 | 0.002 | <0.001 |
| Improved water sources | <--- | Enabling | 0.598 | 0.132 | 0.007 | <0.001 |
| Improved sanitation facilities | <--- | Enabling | 0.055 | 0.013 | 0.005 | 0.012 |
| Seeking treatment | <--- | Utilization | 0.902 | 1.000 |  |  |
| Treatment place | <--- | Utilization | 1.042 | 3.874 | 0.054 | <0.001 |
| ORS use | <--- | Utilization | 0.397 | 0.371 | 0.016 | <0.001 |
| Oral zinc use | <--- | Utilization | 0.355 | 0.303 | 0.015 | <0.001 |

ORS: Oral Rehydration Salts
